# Structural variant calling and clinical interpretation in 6224 unsolved rare disease exomes

**DOI:** 10.1038/s41431-024-01637-4

**Published:** 2024-05-31

**Authors:** German Demidov, Steven Laurie, Annalaura Torella, Giulio Piluso, Marcello Scala, Manuela Morleo, Vincenzo Nigro, Holm Graessner, Siddharth Banka, Solve-RD consortium, German Demidov, Steven Laurie, Annalaura Torella, Giulio Piluso, Marcello Scala, Manuela Morleo, Vincenzo Nigro, Holm Graessner, Siddharth Banka, Alfons Macaya, Belén Pérez-Dueñas, Adam Jackson, Giovanni Stevanin, Jean-Madeleine de Sainte Agathe, Markéta Havlovicová, Rita Horvath, Michele Pinelli, Nienke J. H. van Os, Bart P. C. van de Warrenburg, Anne-Sophie Denommé-Pichon, Marco Savarese, Mridul Johari, Bruno Dallapiccola, Marco Tartaglia, Martje G. Pauly, Anna Katharina Sommer, Tobias B. Haack, Ana Töpf, Lacombe Didier, Chiara Fallerini, Alessandra Renieri, Patrick F. Chinnery, Daniel Natera-de Benito, Andres Nascimento, Aurélien Trimouille, Francina Munell, Anna Marcé-Grau, Ben Yaou Rabah, Gisèle Bonne, Liedewei Van de Vondel, Katja Lohmann, Stephan Ossowski, Katja Lohmann, Stephan Ossowski

**Affiliations:** 1 Institute of Medical Genetics and Applied Genomics, University of Tübingen, Tübingen, Germany; 2 Centro Nacional de Análisis Genómico (CNAG), C/Baldiri Reixac 4, 08028 Barcelona, Spain; 3 Department of Precision Medicine, University of Campania ‘Luigi Vanvitelli’, Naples, Italy; 4 Telethon Institute of Genetics and Medicine, Pozzuoli, Italy; 5 Department of Neurosciences, Rehabilitation, Ophthalmology, Genetics, Maternal and Child Health, Università Degli Studi di Genova, Genoa, Italy; 6 Medical Genetics Unit, IRCCS Istituto Giannina Gaslini, Genoa, Italy; 7 Centre for Rare Diseases, University of Tübingen, Tübingen, Germany; 8 Division of Evolution & Genomic Sciences, School of Biological Sciences, Faculty of Biology, Medicine and Health, University of Manchester, Manchester, UK; 9 Manchester Centre for Genomic Medicine, St. Mary’s Hospital, Manchester University NHS Foundation Trust, Health Innovation Manchester, Manchester, UK; 10 Institute of Neurogenetics, University of Lübeck and University Hospital Schleswig-Holstein, Lübeck, Germany; 11 Pediatric Neurology Research Group, Vall d’Hebron Research Institute, Universitat Autònoma de Barcelona, Barcelona, Spain; 12 Institute of Neuroscience, Universitat Autònoma de Barcelona, Barcelona, Spain; 13 Institut National de la Santé et de la Recherche Medicale (INSERM) U1127, Paris, France; 14 Centre National de la Recherche Scientifique, Unité Mixte de Recherche (UMR) 7225, Paris, France; 15 Unité Mixte de Recherche en Santé 1127, Université Pierre et Marie Curie (Paris 06), Sorbonne Universités, Paris, France; 16 Institut du Cerveau -, ICM Paris, France; 17 Ecole Pratique des Hautes Etudes, Paris Sciences et Lettres Research University, Paris, France; 18 Department of Genetics, Assistance Publique-Hôpitaux de Paris—Sorbonne Université, Pitié-Salpêtrière University Hospital, 83 Boulevard de l’Hôpital, Paris, France; 19 Department of Biology and Medical Genetics, Charles University Prague-2nd Faculty of Medicine and University Hospital Motol, Prague, Czech Republic; 20 Department of Clinical Neurosciences, University of Cambridge, Cambridge, UK; 21 Department of Neurology, Radboud University Medical Center, Nijmegen, The Netherlands; 22 Inserm - University of Burgundy-Franche Comté, UMR1231 GAD, Dijon, France; 23 Folkhälsan Research Centre and Medicum, University of Helsinki, Helsinki, Finland; 24 Molecular Genetics and Functional Genomics, Ospedale Pediatrico Bambino Gesù, IRCCS, Rome, Italy; 25 Institute of Human Genetics, Medical Faculty, University of Bonn, Bonn, Germany; 26 John Walton Muscular Dystrophy Research Centre, Translational and Clinical Research Institute, Newcastle University and Newcastle Hospitals NHS Foundation Trust, Newcastle upon Tyne, UK; 27 Univ. Bordeaux, MRGM INSERM U1211, CHU de Bordeaux, Service de Génétique Médicale, F-33000 Bordeaux, France; 28 Med Biotech Hub and Competence Center, Department of Medical Biotechnologies, University of Siena, Siena, Italy; 29 Medical Genetics, University of Siena, Siena, Italy; 30 Genetica Medica, Azienda Ospedaliero-Universitaria Senese, Siena, Italy; 31 Medical Research Council Mitochondrial Biology Unit, University of Cambridge, Cambridge, UK; 32 Neuromuscular Disorders Unit, Department of Pediatric Neurology. Hospital Sant Joan de Déu, Barcelona, Spain; 33 Laboratoire de Génétique Moléculaire, Service de Génétique Médicale, CHU Bordeaux—Hôpital Pellegrin, Place Amélie Raba Léon, 33076 Bordeaux Cede, France; 34 Sorbonne Université, Inserm, Institut de Myologie, Centre de Recherche en Myologie, F-75013 Paris, France; 35 AP-HP, Centre de Référence de Pathologie Neuromusculaire Nord, Est, Ile-de-France, Institut de Myologie, G.H. Pitié-Salpêtrière, F-75013 Paris, France; 36 Institut de Myologie, Equipe Bases de données, G.H. Pitié-Salpêtrière, F-75013 Paris, France; 37 Peripheral Neuropathy Research Group, University of Antwerp, Antwerp, Belgium; 38 Laboratory of Neuromuscular Pathology, Institute Born-Bunge, University of Antwerp, Antwerpen, Belgium; 39 Translational Neurosciences, Faculty of Medicine and Health Sciences, University of Antwerp, Antwerpen, Belgium

**Keywords:** Genetics research, Data processing

## Abstract

Structural variants (SVs), including large deletions, duplications, inversions, translocations, and more complex events have the potential to disrupt gene function resulting in rare disease. Nevertheless, current pipelines and clinical decision support systems for exome sequencing (ES) tend to focus on small alterations such as single nucleotide variants (SNVs) and insertions-deletions shorter than 50 base pairs (indels). Additionally, detection and interpretation of large copy-number variants (CNVs) are frequently performed. However, detection of other types of SVs in ES data is hampered by the difficulty of identifying breakpoints in off-target (intergenic or intronic) regions, which makes robust identification of SVs challenging. In this paper, we demonstrate the utility of SV calling in ES resulting in a diagnostic yield of 0.4% (23 out of 5825 probands) for a large cohort of unsolved patients collected by the Solve-RD consortium. Remarkably, 8 out of 23 pathogenic SV were not found by comprehensive read-depth-based CNV analysis, resulting in a 0.13% increased diagnostic value.

## Introduction

Next-generation sequencing (NGS) is a method widely used for the detection of single-nucleotide variants (SNVs) and short indels [1] in the clinical diagnosis of rare genetic diseases. Detection of larger or complex variants with short read NGS is challenging as reads with a length of 100–150 bp cannot span across distantly located breakpoints. Nonetheless, methods for the detection of structural variants (SVs), including copy-number variants (CNVs) as well as copy-number neutral variants (such as inversions and balanced translocations) have been developed for genome sequencing (GS). Three main signals are used to detect SVs from genome data: (1) paired-end (PE) orientation and abnormal insert size (distance between read one and read two in a pair), (2) the presence of split and soft-clipped (SC) reads at the breakpoints of SVs, and (3) abnormal read depths (RD) in CNVs [2].

Exome sequencing (ES) is a cost-effective alternative to GS. ES protocols include exon-enrichment by probe hybridization, followed by high-depth sequencing of the enriched exonic regions (typically 100x coverage for clinical exome sequencing). Thus, ES allows reliable detection of small coding and near-splice-site variants within these targeted regions, which are causative for the largest fraction of genetic diseases based on current knowledge. However, depending on the enrichment strategy, ES almost completely lacks coverage of deep-intronic and intergenic variants, making the detection of variants, including SV breakpoints, in these genomic regions essentially impossible. Since it is much more likely for breakpoints of SVs to occur in the >98% non-exonic regions of the human genome, SVs are exceedingly hard to detect with ES data. Hence, usage of PE and SC signals is restricted to breakpoint-detection in regions covered by ES reads [3] and thus, SV detection in ES data is mainly limited to detection of CNVs (deletions and duplications) using the normalized RD signature. It is worth noting that the RD signal in ES suffers from many biases such as GC-content correlated coverage bias and does not allow a robust detection of short coding CNVs affecting only one to several exons, while short CNVs displaying PE or SC signals in addition to changes in RD are more reliably detectable. Therefore, a frequently used approach for SV detection in patients with negative ES results is to perform additional NGS analyses, such as short or long read GS, which increases both cost and time to diagnosis.

Despite the aforementioned issues with detection of SVs in ES data, for approximately 2-4% of SVs (depending on the size of the exome kit’s target region) we would expect the breakpoints to occur close enough to a targeted region, to be detected. In this paper, we evaluate the increase in diagnostic yield by PE- and SC-based SV calling in a large dataset of more than six thousand individuals with rare diseases (Solve-RD data freezes 1 and 2 [4]) who remained undiagnosed after standard ES analysis. Despite the large data heterogeneity, we show a valuable, albeit small increase in the diagnostic yield following SV calling, quality filtering, functional annotation, and clinical interpretation. We demonstrate the benefit of SV calling as part of ES reanalysis for patients with no previously identified molecular cause, underlining the value of applying SV calling algorithms in ES (re-)analysis projects.

## Materials and methods

The recruitment of individuals investigated by the Solve-RD consortium has been described in detail elsewhere [4]. The part of the Solve-RD cohort used in this study includes 9351 exome samples from 9314 individuals (Solve-RD data freezes 1 and 2), including 6224 affected individuals from 5825 families and 3090 unaffected relatives, usually parents. Samples were collected from multiple centers across Europe within four European Reference Networks (ERNs). Our cohort includes (1) 1,892 (30.4%) affected individuals from 1821 families from ERN ITHACA (“Intellectual disability, TeleHealth And Congenital Anomalies”, including 26 affected individuals (0.4%) from 21 families from Undiagnosed Rare Diseases Program (Spain)), (2) 2,343 (37.6%) patients from 2200 families from ERN RND (Rare Neurological Diseases), (3) 1632 (26.2%) patients from 1499 families from EURO-NMD (ERN for NeuroMuscular Diseases) and (4) 357 (5.7%) patients from 340 families from ERN-GENTURIS (GENetic TUmour RIsk Syndromes) (see Laurie et al., 2024, under review, for more details). Patients were submitted by 44 clinical research groups from these 4 ERNs, who used 28 different exome enrichment kits (various vendors and kit versions). Human phenotype ontology (HPO) terms, family relationship information, and raw ES data for all patients were submitted to the RD-Connect GPAP [5] by the corresponding clinicians and submitters.

Reads were aligned to the GRCh37 hs37d5 human reference genome using BWA-MEM 0.7.8. Manta SV caller [6] was used to detect SVs using default parameters and exome flag on. The population allele frequency (AF) of a detected breakpoint was estimated across the complete dataset using the number of breakpoints detected within ±20 base pairs to the focal breakpoint. This vicinity was defined empirically based on the confidence intervals provided by Manta for each detected breakpoint. For approximately 60% of the SVs the confidence intervals are narrower than 40 base pairs, with a median of 17, thus, larger windows would lead to overestimation, while smaller windows would lead to an underestimation of the allele frequency of SVs. Filtering based on breakpoint frequency in the cohort allowed removal of frequently observed events (polymorphisms) and artifacts. We kept only SVs with a breakpoint frequency of less than or equal to 20 out of 9351 exome datasets from affected and unaffected individuals. This AF threshold of ˜0.21% was selected empirically, considering the largest family sizes identified via the relatedness analysis between all samples (largest family has 18 members) and previously reported highest frequencies of variants involved in recessive disorders. Moreover, only variants flagged as “PASSED” by Manta were kept for further interpretation.

Samples submitted by ITHACA, RND, NMD, and GENTURIS were filtered using lists of known disease genes provided by clinical experts from each ERN (3081, 1820, 611, 230 genes, respectively, see Laurie et al. 2024). Only SVs affecting at least one exon or located within 5 bp of an exon of these genes, were reported to the corresponding submitters for clinical interpretation. Intersection of SVs with short HIGH impact variants [7] and known pathogenic missense variants [8] previously dentified in probands, for the identification of compound heterozygous events yielded no additional candidate SVs in disease gene lists. Finally, samples with candidate SVs on more than 5 chromosomes were filtered out, since high numbers of candidate SVs likely suggest low quality of the DNA or sequencing data rather than the presence of several possibly disease-causing rare SVs on different chromosomes. Annotation of SVs reported to clinicians included the following features: chromosome and position of the breakpoints, allele frequency within the Solve-RD exome cohort, affected status of the sequenced individual, HPO terms of the index case, ORDO code, genes potentially affected by the SV, other samples showing the same SV.

Further evaluation of technical quality and potential causality was peformed by clinical researchers from the 42 submitting groups. All SV calls affecting genes reported as autosomal dominant (AD) and X-linked (XL, dominant [XLD] or recessive [XLR]) in OMIM [9] with AF less than 4 per cohort (thus, likely affecting members of only one family or a few independent patients) were evaluated. Investigation of potential biallelic variants in recessive disease genes, i.e., a combination of an SV with a small heterozygous variant on the other allele, was performed by submitters if sufficient expert time was available, which yielded one solved case. Technical validation of calls was undertaken at corresponding facilities. Clear-cut variants were considered as “validation not required”. We define “clear-cut” as events matching all the following criteria: (1) SVs supported by multiple lines of evidence such as normalized coverage depth (CNV calling), split reads, paired-end distance and orientation, and B-allele frequency, (2) phenotypic description (HPO terms) matching to an affected gene, (3) segregation confirmed in all available family members, and (4) visual inspection of the area did not indicate an abundance of “random” split reads or unusual paired end distances, which may indicate some sort of sequencing quality failure. Hence, such SVs required multiple sources of sequencing signal, such as PE and RD in samples with no obvious quality issues, a match of one affected gene with the disease phenotype and the inheritance patterns, as well as no alternative variants explaining the phenotype.

Visual inspection and quality assessment were undertaken using the IGV genome browser. We generated screenshots of the left and right breakpoints and the complete SV as described in [10]. Screenshots accompanying the filtered variants were returned to the data submittersfor inspection.

In addition to Manta, we tested InDelible [11], an SV caller developed specifically for ES data. The suggested pipeline was run according to the authors’ recommendations and further filtered according to the recommended “strict” routine (https://github.com/HurlesGroupSanger/indelible), with the exception of trio-specific filters since most of our samples were singletons. In addition to the author recommended filters, all samples with more than 80,000 detected SVs were filtered out resulting in removal of 7.5% of cases. Results from InDelible were not submitted for expert evaluation, as no additional candidates were obtained, while several good candidates identified by Manta were missed.

Short variant analysis and phenotypic data collection was achieved using the RD-Connect Genome-Phenome Analysis Platform (GPAP) as described in [12]. Parallel RD-based CNV analysis using ClinCNV, ExomeDepth and Conifer was performed as described in [10].

## Results

We detected, quality-filtered, and annotated SVs in 9351 ES datasets collected within the Solve-RD project. SV callsets from unaffected relatives were used for segregation analysis, population-AF based filtering and identification of systematic errors, which helped to dramatically reduce the number of potentially causal SV candidates for in-depth inspection. Following automated quality- and annotation-based filtering of the raw SV callsets generated by Manta, 1404 SV in 868 samples remained in ITHACA, 798 SV in 487 samples in RND, 1519 SV in 606 samples in NMD and 15 SV in 15 samples in GENTURIS callsets. The distribution of SVs per sample was not uniform, and some samples contained a high number of candidates for clinical interpretation.

Upon expert-inspection by experts from the corresponding European Research Networks participating in Solve-RD, 23 distinct SVs were considered to be causal in 32 out of 6,224 (0.51%) affected individuals, pertaining to 23 unrelated families (Table 1). Eight of these 23 SVs (0.13% of the affected individuals) were not reported by read depth (RD) based CNV detection using ClinCNV [13], ExomeDepth [14] and Conifer [15] performed in parallel [10] (Table 2). To evaluate the added diagnostic value in comparison with conventional read-depth CNV analysis for ES, we present the identified SVs in three categories.

**Table 1:** The total number of structural variant calls, the number of evaluated calls and the diagnostic value increase per ERN. Total number of affected individuals denotes all the affected family members including index cases.

	ERN RND	ERN ITHACA	ERN NMD	ERN GENTURIS
Number of affected individuals	2.343	1.892	1.632	357
Number of index patients	2.2	1.821	1.499	340
Known disease genes in gene list	1.82	3.081	611	230
Number of candidate variants, after filtering	798	1.404	1.519	15
Number of samples with SVs, after filtering	487	868	606	15
Number of solved index patients/all affected patients	7 (0.32%) / 11	9 (0.49%) / 9	6 (0.4%) / 9	1 (0.29%) / 3
Percentage of causal SVs among investigated SVs	1.37%	0.64%	0.59%	20%

**Table 2:**
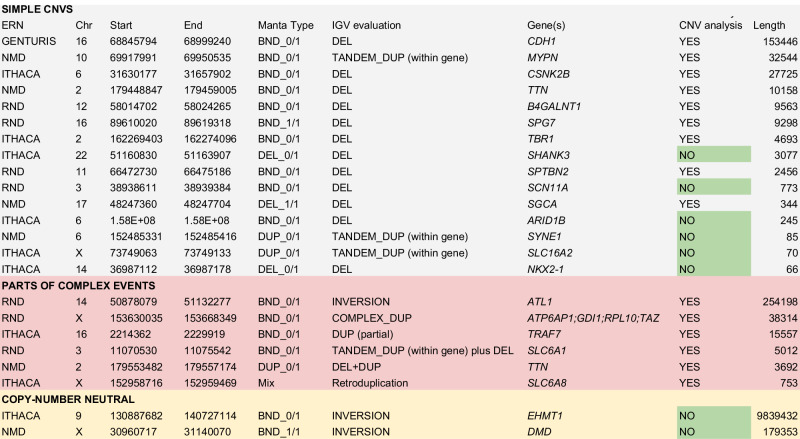
Variants detected via paired-end or soft-clipped signal based SV analysis (Manta) in exomes, considered to be causative for the corresponding rare diseases.

First, a detected SV can be classified as a simple deletion or duplication (CNV). 15 distinct SVs in 19 patients were simple CNVs, however, 5 of them were not detected by the CNV detection approaches due to their small size (typicallyaffecting a single exon or only part of an exon affected by deletions with overall length ranging from 66 to 3077 base pairs). The longest CNV, not detected by RD methods, was a 3077 base-pair long deletion, which only affects 36 base pairs of exon 23 of *SHANK3* (NM_001372044.1), an exon with a total length of 2.2 kb. One simple deletion affecting the recessive gene *B4GALNT1* was not reported in RD-based CNV analysis due to an excessive number of CNV candidates in the sample, but was prioritized in our SV analysis, which produced a much smaller number of calls for expert evaluation. Further inspection of this 9563 bp deletion, removing six exons, revealed that it was detected by RD-based CNV callers. Moreover, a missense variant, known to be pathogenic, was detected in the other allele, thus forming a compound heterozygote explaining the phenotype [Laurie at al 2024, under review]. It is worth mentioning that even for SVs detected in parallel by RD-based CNV callers the accurate information on breakpoint coordinates can facilitate the functional interpretation of the variant, as in e.g. [16].

The second category comprises variants for which a detected SV can be a part of a complex SV (a combination of deletions, duplications, and inversions), part of which is detectable via standard RD-based CNV analysis. It is rarely possible to detect all breakpoints in this situation using ES data. However, detected SVs in combination with CNVs detected by RD-based methods can indicate and potentially better resolve the complex nature of an event. Six variants which were considered causal in 10 patients were complex events, such as a deletion followed by a duplication, or an inversion followed by a duplication.

**Fig. 1:**
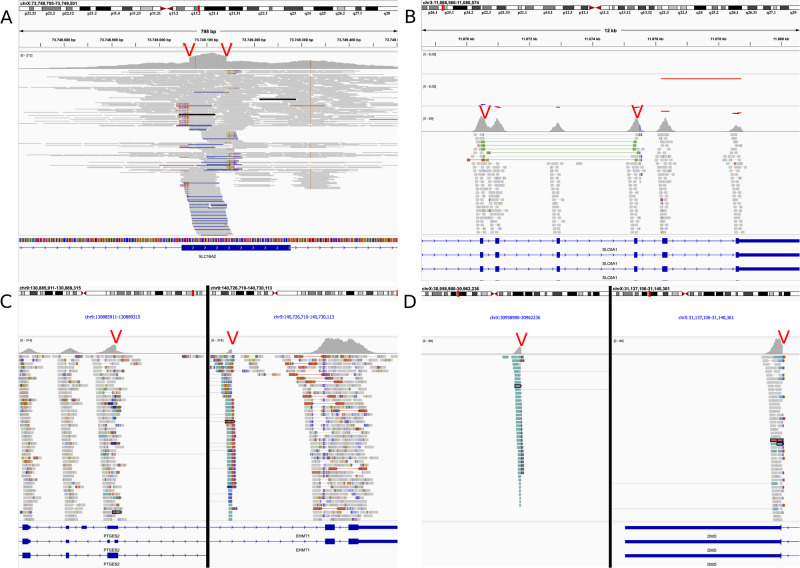
Visualisation of read aligments for various types of pathogenic SVs in the tool IGV. **A** Simple duplication of 70 base pairs missed by RD-based CNV analysis and short variant calling. **B** A complex SV: paired-end distance indicates the presence of a tandem duplication. RD-based CNV calling (top 2 tracks) indicates the presence of a deletion next to the detected duplication, together forming a complex SV. **C** A 9.8Mb- long genomic inversion affecting the penultimate intron in *EHMT1* was detected via structural variant calling. **D** 179Kb long inversion involving the last exon of the DMD gene. Screenshots produced using Integrative Genomics Viewer [21]. Red arrows indicate the breakpoints identified via SV analysis.

Finally, a detected SV can be copy-number neutral. We were able to find causal inversions, but no translocations. Two pathogenic inversions were found in two patients. The first one presented an inversion of almost 10 Mb in length affecting *EHMT1* (MIM * 607001) in intron 25 of 26 (g:9:130,887,682-140,727,115, hg19) (ERN-ITHACA; Fig. 1C) This subject meets the criteria for Kleefstra syndrome type 1 (KS1) [17], a well-described syndromic neurodevelopmental condition characterized by psychomotor delay, cognitive impairment, behavioral disorders, facial dysmorphism, abnormal skull shape, abnormalities of hands, and congenital heart defects [17, 18]. Indeed, this patient presented with severe intellectual disability including absence of speech, hand stereotypies, aggressivity, and hypotonia. Additional clinical manifestations consistent with a possible diagnosis of KS1 included dysmorphic features (e.g., sparse eyebrows, short nose, protruding tongue, absence of lateral incisors) and hand abnormalities (syndactyly and drumstick fingers). Considering the suggestive phenotype, genetic testing for KS1 had previously been performed through array comparative genomic hybridization (aCGH) and Sanger sequencing of the *EHMT1* gene, according to the suggestions from the diagnostic guidelines [17]. However, these tests did not detect any possibly pathogenic variant in the candidate gene, leading to several years of a delay in diagnosing the patient despite the strongly suggestive clinical and phenotypical observations.Following the identification of the 10 Mb inversion identified here, karyotyping and FISH were performed to furtherly validate the molecular diagnosis. Karyotyping at 400 band resolution did not identify the inversion that was correctly observed by FISH (probe utilized RP11-31M4 (9q34.3) Empire Genomics) (data not shown). The second patient had an inversion of approximately 180 kb in length affecting *DMD* (ERN-NMD; Fig. 1D). Both inversions occurred in patients with strikingly fitting phenotypes which had been extensively examined for variants in the corresponding genes as candidate genes for several years before being enrolled into the Solve-RD project. The genes are directly affected by these inversions, leading to loss-of-function phenotypes.

Thus, the added diagnostic value gained solely from paired-end mapping-based SV detection in the reanalysis of unsolved individuals, was 8 out of 5825 index patients (0.14%) for pipelines that include a comprehensive read-depth based CNV detection method. This value increases to 0.4% (23/5,825) for pipelines detecting only short variants, hence lacking CNV calls.

In addition to Manta, we initially tested InDelible [11], an SV caller developed specifically for ES data. Despite the lower allele frequency filtering threshold, recommended by the authors (0.04% in comparison with 0.2% we used in our analysis), 684 variants passed the filtering (Manta: 1976, Table 1). A preliminary examination of the calls revealed no additional candidates not reported by Manta, but InDelible missed all the clear rare candidates detectable with paired-end information. Thus, InDelible results were not submitted for expert evaluation and were excluded from further use in this study.

## Discussion

Here we demonstrate the utility of PE- and SC-based SV calling in ES data for undiagnosed individuals with a rare disease using a large cohort of almost 10,000 individuals from the Solve-RD project. Despite the modest overall increase in the diagnostic yield, each successfully diagnosed patient represents a family whose diagnostic odyssey is coming to an end. Especially for patients initially sequenced many years ago, alternative ways of investigating structural variants, such as GS or long-read sequencing, may be unavailable due to financial or logistical reasons e.g. lack of DNA samples. Thus, re-analysis approaches using existing ES data to solve unsolved patients can benefit from PE-based SV calling. In a global effort to solve previously unsolved patients, SV calling in ES data could help hundreds of people to find their diagnosis after many years of being undiagnosed.

Even though the majority of our findings (15 out of 23 unique causal SVs) were also discovered in parallel via read-depth based CNV analysis, SV identification using PE and SC signals should not be considered redundant in such cases. In some patients (6 out of 23 variants) the detected SVs occur next to CNVs detected by RD callers, but knowledge of the SV’s presence is crucial for the interpretation of the rearrangement, uncovering its underlying complexity, and may indicate the necessity for additional molecular analyses such as long read DNA sequencing or RNA sequencing. Furthermore, SV detection in ES, even when it identifies the same variant also found by RD methods, increases the confidence that the variant is a true positive. PE-based SV calling facilitates the validation of CNVs since it provides information about the exact breakpoint coordinates and enables locus-specific PCR amplification and Sanger sequencing. Moreover, we were able to identify eight SVs that were not identified by RD methods [10], five of which were simple deletions and duplications, two were inversions, and one was a larger deletion forming a compound heterozygote with a SNV in an autosomal recessive disease gene (which was not reported based on RD-calling due to an excessive number of CNV calls in recessive genes).

Notably, both inversions were identified in patients with strikingly matching phenotypes, who had undergo multiple rounds of genetic testing of the respective genes before the start of Solve-RDIn this regard, the reanalysis of the genetic data focused on the investigation of structural variants (SV) through the Solve-RD platform was fundamental for the identification of a balanced rearrangement causing the disruption of *EHMT1*, thus providing the pathophysiological link to the genetic condition displayed by the proband of this family. This achievement ended the diagnostic odyssey of this family, finally offering a genetic diagnosis and the chance of targeted clinical management.

In comparison with widely used RD based CNV callers, we cannot estimate the recall rate for detecting variants with PE-based SV calling from ES data, since breakpoints need to affect a targeted region of the exome. For this reason, the chance to successfully identify an SV is independent of its length, as its breakpoints must be covered with a significant number of short reads exhibiting abnormal orientation or insert size. Nonetheless, among our more than 6000 index patients, five CNVs were detected that were missed by CNV analysis since they were too short. Furthermore, RD-based CNV-calling cannot detect copy-number-neutral events, such as two inversions in our cohort. 16 out of 105 unique CNVs identified as being pathogenic or likely-pathogenic [10] were also detected in parallel by SV analysis (24 out of 115 solved, candidate or partially explaining the phenotype patients), allowing to better define their breakpoints. Interestingly, this fraction is higher than expected when considering the genomic region covered by ES ( < 2%), likely because we focus our analysis on SVs and CNVs overlapping known disease genes. Moreover, the chance of detected CNVs to be true positives increases, if they are supported by paired-end and split-read signatures, allowing genetic analysts to evaluate the clinical relevance of the variant without doubts regarding the technical quality. Finally, CNVs detected in ES data do not provide accurate breakpoint information as SV calling can, which further simplifies the clinical interpretation of variants. Hence, we conclude that SV calling in ES often provides valuable additional information even for variants already detected by CNV analysis.

A previously published study by [11] in which SVs were analyzed in a cohort of 13,438 probands from the Deciphering Developmental Disorders (DDD) study came to comparable conclusions regarding the fraction of detectable causal SVs. Gardner et al. used a combination of the tool XHMM [19] for RD-based CNV calling and InDelible for split read (SR)-based SV calling, while we combined three RD-based tools with the SV caller Manta. Gardner et al. report 30 unique pathogenic SVs identified by InDelible (0.22%) in addition to 128 CNVs detected by XHMM (0.95%). Interestingly, the fraction of causal CNVs found via the RD-method XHMM is substantially lower compared to the combination of three RD-Methods in Solve-RD (0.95% vs. 1.6%) [10], but on the other hand the additional diagnostic value of SVs is slightly higher (0.22% vs. 0.14% in our study). Of note, applying InDelible to our cohort (data not shown) did not reveal additional SVs except for 3 mobile element insertions (MEI), which we had already detected using specialized tools [20], but it did miss several causal SVs identified by Manta. We conclude that despite using different patient cohorts and different approaches for the detection of SVs and CNVs, the DDD and Solve-RD initiatives produced comparable results regarding the fraction of causal SVs detected.

The categorization of the identified variants into three groups also allows us to specify the technical and analytical challenges encountered while evaluating them. For the first group (simple deletions and duplications) the main challenge was to evaluate the technical quality of the calls which were missed by RD methods. In these cases, we had to rely only on paired-end and split-read information, since the coverage did not change visibly beyond the standard level of noise. The evaluation of the SVs that we defined as “complex” necessarily involved joint visual analysis of RD and PE signals. Since we could often only detect one pair of breakpoints in “complex” SVs, only the presence of abnormal coverage next to the identified SV indicated the complex nature of the event. In the case of inversions, in most cases Manta did not report the type of variant as INV (inversion) but BND (breakpoint) in ES data, and hence all breakpoints had to be visually explored in order to identify inversions. This weakness can only be overcome by genome sequencing, which will typically result in clear identification of an inversion.

As discussed, the PE-based SV calling approach using ES has some weaknesses. We did not perform an evaluation of SV calling sensitivity in ES since the sensitivity is low, as expected by the nature of targeted sequencing. Usage of Manta or analogous tools for SV detection cannot replace RD-based CNV detection. SV calling should only be considered as an addition, which may result in a slight increase indiagnostic yield but does not guarantee robust detection even of long rearrangements if their breakpoints are intergenic or deep-intronic. Furthermore, improvements and automation of the clinical interpretation of SVs occurring in recessive genes are necessary to reduce the number of reported calls for expert evaluation, as all tools report many false positives. One way to achieve this is through automated phenotypic matching procedures, which we plan to implement during the analysis of the final Solve-RD data freeze and will report the results to the medical genetics community.

In summary, PE and SC based SV calling is a valuable addition to RD-based CNV calling, providing a diagnosis to a small but important fraction of rare disease patients, who would otherwise remain undiagnosed.

## Supplementary information

Supplementary Material

## Data Availability

All raw and processed data files are available at the EGA (Datasets EGAD00001009767, EGAD00001009768, EGAD00001009769, and EGAD00001009770, under Solve-RD study EGAS00001003851). The family (FAM) and participant (P) identifiers used in this manuscript are pseudonymized and known only to the researchers involved In Solve-RD.
