# Supplementary material for: Structural variant calling and clinical interpretation in 6224 unsolved rare disease exomes
Source: Eur J Hum Genet. 2024 May 31;32(8):998–1004. doi: 10.1038/s41431-024-01637-4 (PMC11291474; doi:10.1038/s41431-024-01637-4)
Supplement: Supplementary file 1 — Supplementary Material [file 41431_2024_1637_MOESM1_ESM.docx]

**Solve-RD consortium**

**EKUT:** Olaf Riess^1, 2^, Tobias B. Haack^1^, Holm Graessner^1, 2^, Birte Zurek^1, 2^, Kornelia Ellwanger^1, 2^, Stephan Ossowski^1, 3^, German Demidov^1^, Marc Sturm^1^, Julia M. Schulze-Hentrich^1^, Rebecca Schüle^1, 2^, Jishu Xu^4, 5^, Christoph Kessler^4, 5^, Melanie Kellner^4, 5^, Matthis Synofzik^4, 5^, Carlo Wilke^4, 5^, Andreas Traschütz^4, 5^, Ludger Schöls^4, 5^, Holger Hengel^4, 5^, Holger Lerche^1^, Josua Kegele^6^, Peter Heutink^4, 5^

**RUMC:** Han Brunner^7-9^, Hans Scheffer^7, 8^, Nicoline Hoogerbrugge^7, 10^, Alexander Hoischen^7, 10, 11^, Peter A.C. ’t Hoen ^10, 12^, Lisenka E.L.M. Vissers^7, 8^, Christian Gilissen^7, 10^, Wouter Steyaert^7, 10^, Karolis Sablauskas^7^, Richarda M. de Voer^7, 10^, Erik-Jan Kamsteeg^7^, Bart van de Warrenburg^8, 13^, Nienke van Os^8, 13^, Iris te Paske^7, 10^, Erik Janssen^7, 10^, Elke de Boer^7, 8^,Marloes Steehouwer^7^, Burcu Yaldiz^7^, Tjitske Kleefstra^7, 8^

**University of Leicester**: Anthony J. Brookes^14^, Colin Veal^14^, Spencer Gibson^14^, Vatsalya Maddi^14^, Mehdi Mehtarizadeh^14^, Umar Riaz^14^, Greg Warren^14^, Farid Yavari Dizjikan^14^, Thomas Shorter^14^

**UNEW:** Ana Töpf^15^, Volker Straub^15^, Chiara Marini Bettolo^15^, Jordi Diaz Manera^15^, Sophie Hambleton^16^, Karin Engelhardt^16^

**MUH:** Jill Clayton-Smith^17, 18^, Siddharth Banka^17, 18^, Elizabeth Alexander^18^, Adam Jackson^17, 18^

**DIJON:** Laurence Faivre^19-23^, Christel Thauvin^19-23^, Antonio Vitobello^21^, Anne-Sophie Denommé-Pichon^21^, Yannis Duffourd^21, 22^, Ange-Line Bruel^21^, Christine Peyron^24, 25^, Aurore Pélissier^24, 25^

**CNAG-CRG:** Sergi Beltran^26, 27^, Ivo Glynne Gut^26, 27^, Steven Laurie^26^, Davide Piscia^26^, Leslie Matalonga^26^, Anastasios Papakonstantinou^26^, Gemma Bullich^26^, Alberto Corvo^26^, Marcos Fernandez-Callejo^26^, Carles Hernández^26^, Daniel Picó^26^, Ida Paramonov^26^, Hanns Lochmüller^26^

**EURORDIS:** Gulcin Gumus^28^, Virginie Bros-Facer^29^

**INSERM-Orphanet:** Ana Rath^30^, Marc Hanauer^30^, David Lagorce^30^,Oscar Hongnat^30^,Maroua Chahdil^30^,Emeline Lebreton^30^

**INSERM-ICM:** Giovanni Stevanin^31-35^, Alexandra Durr^31-34, 36^, Claire-Sophie Davoine^31-35^, Léna Guillot-Noel^31-35^, Anna Heinzmann ^31-34, 37^, Giulia Coarelli^31-34, 37^

**INSERM-CRM:** Gisèle Bonne^38^, Teresinha Evangelista^38^, Valérie Allamand^38^, Isabelle Nelson^38^, Rabah Ben Yaou^38-40^, Corinne Metay^38, 41^, Bruno Eymard^38, 39^, Enzo Cohen^38^, Antonio Atalaia^38^, Tanya Stojkovic^38, 39^

**Univerzita Karlova:** Milan Macek Jr.^42^, Marek Turnovec^42^, Dana Thomasová^42^, Radka Pourová Kremliková^42^, Vera Franková^42^, Markéta Havlovicová^42^, Petra Lišková^43, 44^, Pavla Doležalová^45^

**EMBL-EBI:** Helen Parkinson^46^, Thomas Keane^46^, Mallory Freeberg^46^, Coline Thomas^46^, Dylan Spalding^46^

**Jackson Laboratory**: Peter Robinson^47^, Daniel Danis^47^

**KCL**: Glenn Robert^48^, Alessia Costa^49^, Christine Patch^49, 50^

**UCL-IoN**: Mike Hanna^51^, Henry Houlden^52^, Mary Reilly^51^, Jana Vandrovcova^52^, Stephanie Efthymiou^52^, Heba Morsy^52^, Elisa Cali^52^, Francesca Magrinelli^53^, Sanjay M. Sisodiya^54^, Jonathan Rohrer^55^

**UCL-ICH**, Francesco Muntoni^56, 57^, Irina Zaharieva^56^, Anna Sarkozy^56^

**Universiteit Antwerpen**: Vincent Timmerman^58, 59^, Jonathan Baets^60, 61^, Geert de Vries^59, 60^, Jonathan De Winter^59-61^, Danique Beijer^58-60^, Peter de Jonghe^59, 61^, Liedewei Van de Vondel^58-60^, Willem De Ridder^59-61^, Sarah Weckhuysen^60, 62^

**Uni Naples/Telethon UDP**: Vincenzo Nigro^63, 64^, Margherita Mutarelli^64, 65^, Manuela Morleo^64^, Michele Pinelli^64^, Alessandra Varavallo^64^, Sandro Banfi^63, 64^, Annalaura Torella^63^, Francesco Musacchia^63, 64^, Giulio Piluso^63^

**UNIFE**: Alessandra Ferlini^66^, Rita Selvatici^66^, Francesca Gualandi^66^, Stefania Bigoni^66^, Rachele Rossi^66^, Marcella Neri^66^

**UKB**: Stefan Aretz^67, 68^, Isabel Spier^67, 68^, Anna Katharina Sommer^67^, Sophia Peters^67^

**IPATIMUP**: Carla Oliveira^69-71^, Jose Garcia-Pelaez^69, 70, 72^, Rita Barbosa**-**Matos^69, 70, 73^, Celina São José^69, 70, 72^ , Marta Ferreira^69, 70, 74^, Irene Gullo^69-71, 75^, Susana Fernandes^76^, Luzia Garrido^75^, Pedro Ferreira^69, 70, 77^, Fátima Carneiro^69-71, 75^

**UMCG**: Morris A Swertz^78^, Lennart Johansson^78^, Joeri K van der Velde^78^, Gerben van der Vries^78^, Pieter B Neerincx^78^, David Ruvolo^78^, Kristin M Abbott^79^, Wilhemina S Kerstjens Frederikse^79, 80^, Eveline Zonneveld-Huijssoon^79, 81^, Dieuwke Roelofs-Prins^78^, Marielle van Gijn^79, 81^

**Charité**: Sebastian Köhler^82^

**SHU**: Alison Metcalfe^48, 83^

**APHP**: Alain Verloes^84, 85^, Séverine Drunat^84, 85^, Delphine Heron^86, 87^, Cyril Mignot^86, 88^, Boris Keren^86^, Jean-Madeleine de Sainte Agathe^86^

**CHU Bordeaux**: Caroline Rooryck^89^, Didier Lacombe^89^, Aurelien Trimouille^90^

**Spain UDP**: Manuel Posada De la Paz^91^, Eva Bermejo Sánchez^91^, Estrella López Martín^91^, Beatriz Martínez Delgado^91^, F. Javier Alonso García de la Rosa^91^

**Ospedale Pediatrico Bambino Gesù, Rome**: Andrea Ciolfi^92^, Bruno Dallapiccola^92^, Simone Pizzi^92^, Francesca Clementina Radio^92^, Marco Tartaglia^92^

**University of Siena**: Alessandra Renieri^93-95^, Simone Furini^93, 94^, Chiara Fallerini^93, 94^, Elisa Benetti^93, 94^

**Semmelweis University Budapest**: Peter Balicza^96^, Maria Judit Molnar^96^

**University of Ljubljana**, Ales Maver^97^, Borut Peterlin^97^

**University of Lübeck**: Alexander Münchau^98^, Katja Lohmann^99^, Rebecca Herzog^98, 100^, Martje Pauly^98, 99^

**Val d'Hebron Barcelona**: Alfons Macaya^101, 102^, Ana Cazurro-Gutiérrez^101^, Belén Pérez-Dueñas^101^, Francina Munell^101^, Clara Franco Jarava^103, 104^, Laura Batlle Masó^105, 106^, Anna Marcé-Grau^101^, Roger Colobran^103, 104, 107^

**Hospital Sant Joan de Déu Barcelona**: Andrés Nascimento Osorio^108^, Daniel Natera de Benito^108^

**University of Freiburg**: Hanns Lochmüller^109-111^, Rachel Thompson^111^, Kiran Polavarapu^111^, Bodo Grimbacher^112-116^

**University of Oxford**: David Beeson^117^, Judith Cossins^117^

**Folkhälsan Research Centre**: Peter Hackman^118^, Mridul Johari^118^, Marco Savarese^118^, Bjarne Udd^118-120^

**University of Cambridge**: Rita Horvath^121^, Patrick F. Chinnery^121, 122^, Thiloka Ratnaike^123^, Fei Gao^121^, Katherine Schon^121, 124^

**Catalan Institute of Oncology, Barcelona**: Gabriel Capella^125^, Laura Valle^125^

**KU Munich**: Elke Holinski-Feder^126^, Andreas Laner^127^, Verena Steinke-Lange^126^

**TU Dresden**: Evelin Schröck^128^, Andreas Rump^128, 129^

**Koç University:** Ayşe Nazlı Başak^130^

**Ghent University Hospital**: Dimitri Hemelsoet^131, 132^, Bart Dermaut^132-134^, Nika Schuermans^132-134^, Bruce Poppe^132-134^, Hannah Verdin^133^

**University Hospital Meyer, Florence**: Davide Mei^135^, Annalisa Vetro^135^, Simona Balestrini^135, 136^, Renzo Guerrini^135^

**KU Leuven**: Kristl Claeys^137, 138^

**LUMC**: Gijs W.E. Santen^139^, Emilia K. Bijlsma^139^, Mariette J.V. Hoffer^139^, Claudia A.L. Ruivenkamp^139^

**Ludwig Boltzmann Institute for Rare and Undiagnosed Diseases, Vienna:** Kaan Boztug^140-144^, Matthias Haimel^140-142^

**Institute of Pathology and Genetics, Gosselies, Belgium**: Isabelle Maystadt^145, 146^

**Technical University Munich**: Isabell Cordts^147^, Marcus Deschauer^147^

**Neurology/Neurogenetics Laboratory University of Crete, Heraklion, Crete, Greece:** Ioannis Zaganas^148^, Evgenia Kokosali^148^, Mathioudakis Lambros^148^, Athanasios Evangeliou^149^, Martha Spilioti^150^, Elisabeth Kapaki^151^, Mara Bourbouli^151^

**IRCCS G. Gaslini**: Pasquale Striano^152, 153^, Federico Zara^153, 154^, Antonella Riva^153, 154^, Michele Iacomino^154, 155^, Paolo Uva^155^, Marcello Scala^152, 153^, Paolo Scudieri^153, 154^

**Cliniques universitaires Saint-Luc (CUSL)**: Maria-Roberta Cilio^156^, Evelina Carpancea^156^, Chantal Depondt^157^, Damien Lederer^158^, Yves Sznajer^159^, Sarah Duerinckx^160^, Sandrine Mary^158^

**Institute of Human Genetics, University Hospital Essen**: Christel Depienne^161, 162^, Andreas Roos^111, 163, 164^

**University of Luxembourg**: Patrick May^165^

**Affiliations**

1. Institute of Medical Genetics and Applied Genomics, University of Tübingen, Tübingen, Germany.

2. Centre for Rare Diseases, University of Tübingen, Tübingen, Germany.

3. NGS Competence Center Tübingen (NCCT), University of Tübingen, Tübingen, Germany.

4. Department of Neurodegeneration, Hertie Institute for Clinical Brain Research (HIH), University of Tübingen, Tübingen, Germany.

5. German Center for Neurodegenerative Diseases (DZNE), Tübingen, Germany.

6. Department of Neurology and Epileptology, Hertie Institute for Clinical Brain Research (HIH), University of Tübingen, Tübingen, Germany.

7. Department of Human Genetics, Radboud University Medical Center, Nijmegen, The Netherlands.

8. Donders Institute for Brain, Cognition and Behaviour, Radboud University Medical Center, Nijmegen, The Netherlands.

9. Department of Clinical Genetics, Maastricht University Medical Centre, Maastricht, the Netherlands.

10. Radboud Institute for Molecular Life Sciences, Nijmegen, The Netherlands.

11. Department of Internal Medicine and Radboud Center for Infectious Diseases (RCI), Radboud University Medical Center, Nijmegen, the Netherlands.

12. Center for Molecular and Biomolecular Informatics, Radboud University Medical Center, Nijmegen, the Netherlands.

13. Department of Neurology, Radboud University Medical Center, Nijmegen, The Netherlands.

14. Department of Genetics and Genome Biology, University of Leicester, Leicester, UK.

15. John Walton Muscular Dystrophy Research Centre, Translational and Clinical Research Institute, Newcastle University and Newcastle Hospitals NHS Foundation Trust, Newcastle upon Tyne, UK.

16. Primary Immunodeficiency Group, Translational and Clinical Research Institute, Newcastle University and Newcastle upon Tyne Hospitals NHS Foundation Trust, Newcastle upon Tyne, UK.

17. Division of Evolution, Infection and Genomics, School of Biological Sciences, Faculty of Biology, Medicine and Health, University of Manchester, Manchester M13 9WL, UK.

18. Manchester Centre for Genomic Medicine, St Mary's Hospital, Manchester University Hospitals NHS Foundation Trust, Health Innovation Manchester, Manchester M13 9WL, UK.

19. Dijon University Hospital, Genetics Department, Dijon, France.

20. Dijon University Hospital, Centre of Reference for Rare Diseases: Development disorders and malformation syndromes, Dijon, France.

21. Inserm - University of Burgundy-Franche Comté, UMR1231 GAD, Dijon, France.

22. Dijon University Hospital, FHU-TRANSLAD, Dijon, France.

23. Dijon University Hospital, GIMI institute, Dijon, France.

24. University of Burgundy-Franche Comté, Dijon Economics Laboratory, Dijon, France.

25. University of Burgundy-Franche Comté, FHU-TRANSLAD, Dijon, France.

26. CNAG-CRG, Centre for Genomic Regulation (CRG), The Barcelona Institute of Science and Technology, Baldiri Reixac 4, Barcelona 08028, Spain.

27. Universitat Pompeu Fabra (UPF), Barcelona, Spain.

28. EURORDIS-Rare Diseases Europe, Sant Antoni Maria Claret 167 - 08025 Barcelona, Spain.

29. EURORDIS-Rare Diseases Europe, Plateforme Maladies Rares, 75014 Paris, France.

30. INSERM, US14 - Orphanet, Plateforme Maladies Rares, 75014 Paris, France.

31. Institut National de la Santé et de la Recherche Medicale (INSERM) U1127, Paris, France.

32. Centre National de la Recherche Scientifique, Unité Mixte de Recherche (UMR) 7225, Paris, France.

33. Unité Mixte de Recherche en Santé 1127, Université Pierre et Marie Curie (Paris 06), Sorbonne Universités, Paris, France.

34. Institut du Cerveau - ICM, Paris, France.

35. Ecole Pratique des Hautes Etudes, Paris Sciences et Lettres Research University, Paris, France.

36. Centre de Référence de Neurogénétique, Hôpital de la Pitié-Salpêtrière, Assistance Publique-Hôpitaux de Paris (AP-HP), Paris, France.

37. Hôpital de la Pitié-Salpêtrière, Assistance Publique-Hôpitaux de Paris (AP-HP), Paris, France.

38. Sorbonne Université, Inserm, Institut de Myologie, Centre de Recherche en Myologie, F-75013 Paris, France.

39. AP-HP, Centre de Référence de Pathologie Neuromusculaire Nord, Est, Ile-de-France, Institut de Myologie, G.H. Pitié-Salpêtrière, F-75013 Paris, France.

40. Institut de Myologie, Equipe Bases de données, G.H. Pitié-Salpêtrière, F-75013 Paris, France.

41. AP-HP, Unité Fonctionnelle de Cardiogénétique et Myogénétique Moléculaire et Cellulaire, G.H. Pitié-Salpêtrière, F-75013 Paris, France.

42. Department of Biology and Medical Genetics, Charles University Prague-2nd Faculty of Medicine and University Hospital Motol, Prague, Czech Republic.

43. Department of Paediatrics and Inherited Metabolic Disorders, First Faculty of Medicine, Charles University and General University Hospital in Prague, Prague, Czech Republic.

44. Department of Ophthalmology, First Faculty of Medicine, Charles University and General University Hospital in Prague, Prague, Czech Republic.

45. Centre for Paediatric Rheumatology and Autoinflammatory Diseases, Department of Paediatrics and Inherited Metabolic Disorders, 1st Faculty of Medicine, Charles University and General University Hospital in Prague, Czech Republic.

46. European Bioinformatics Institute, European Molecular Biology Laboratory, Wellcome Genome Campus, Hinxton, Cambridge, United Kingdom.

47. Jackson Laboratory for Genomic Medicine, Farmington, CT 06032, USA.

48. Florence Nightingale Faculty of Nursing, Midwifery & Palliative Care, King's College, London, UK.

49. Society and Ethics Research, Connecting Science, Wellcome Genome Campus,

Hinxton, UK.

50. Genomics England, Queen Mary University of London, Dawson Hall, EC1M 6BQ, London, UK.

51. MRC Centre for Neuromuscular Diseases and National Hospital for Neurology and Neurosurgery, UCL Queen Square Institute of Neurology, London, UK.

52. Department of Neuromuscular Diseases, UCL Queen Square Institute of Neurology, London, UK.

53. Department of Clinical and Movement Neurosciences, UCL Queen Square Institute of Neurology, University College London, WC1N 3BG.

54. Department of Clinical and Experimental Epilepsy, UCL Queen Square Institute of Neurology, London, UK.

55. Dementia Research Centre, Department of Neurodegenerative Disease, UCL Queen Square Institute of Neurology, London, UK.

56. Dubowitz Neuromuscular Centre, UCL Great Ormond Street Hospital, London, UK.

57. NIHR Great Ormond Street Hospital Biomedical Research Centre, London, United Kingdom.

58. Peripheral Neuropathy Research Group, University of Antwerp, Antwerp, Belgium.

59. Laboratory of Neuromuscular Pathology, Institute Born-Bunge, University of Antwerp, Antwerpen, Belgium.

60. Translational Neurosciences, Faculty of Medicine and Health Sciences, University of Antwerp, Belgium.

61. Neuromuscular Reference Centre, Department of Neurology, Antwerp University Hospital, Antwerpen, Belgium.

62. VIB-CMN, Applied and Translational Neurogenomics Group.

63. Dipartimento di Medicina di Precisione, Università degli Studi della Campania "Luigi Vanvitelli", Napoli, Italy.

64. Telethon Institute of Genetics and Medicine, Pozzuoli, Italy.

65. Istituto di Scienze Applicate e Sistemi Intelligenti "E.Caianiello" - ISASI -CNR.

66. Unit of Medical Genetics, Department of Medical Sciences, University of Ferrara, Italy.

67. Institute of Human Genetics, Medical Faculty, University of Bonn, Bonn, Germany.

68. Center for Hereditary Tumor Syndromes, University Hospital Bonn, Bonn, Germany.

69. i3S - Instituto de Investigação e Inovação em Saúde, Universidade do Porto, Portugal.

70. IPATIMUP - Institute of Molecular Pathology and Immunology of the University of Porto, Portugal.

71. Faculty of Medicine, University of Porto, Portugal.

72. Doctoral Programme in Biomedicine, Faculty of Medicine, University of Porto, Portugal.

73. Doctoral Programme in BiotechHealth, School of Medicine and Biomedical Sciences, University of Porto, Portugal.

74. Doctoral Programme in Computer Science, Faculty of Sciences, University of Porto, Portugal.

75. CHUSJ, Centro Hospitalar e Universitário de São João, Porto, Portugal.

76. Departament of Genetics, Faculty of Medicine, University of Porto, Portugal.

77. Faculty of Sciences, University of Porto, Portugal.

78. Department of Genetics, Genomics Coordination Center, University Medical Center Groningen, University of Groningen, Groningen, The Netherlands.

79. Department of Genetics, University Medical Center Groningen, University of Groningen, Groningen, The Netherlands.

80. ERN-GENTURIS.

81. ERN-RITA: European Reference Network for Immunodeficiency, Autoinflammatory, Autimmune and Paediatric Rheumatic diseases, Utrecht, Netherlands.

82. Ada Health GmbH, Karl-Liebknecht-Str. 1, 10178 Berlin, Germany.

83. College of Health, Well-being and Life-Sciences, Sheffield Hallam University, Sheffield, UK.

84. Dept of Genetics, Assistance Publique-Hôpitaux de Paris - Université de Paris, Robert DEBRE University Hospital, 48 bd SERURIER, Paris, France.

85. INSERM UMR 1141 "NeuroDiderot", Hôpital Robert DEBRE, Paris, France.

86. Department of Genetics, Assistance Publique-Hôpitaux de Paris - Sorbonne Université, Pitié-Salpêtrière University Hospital, 83 Boulevard de l'Hôpital, Paris, France.

87. Reference center of rare diseases "intellectuel disability of rare causes", Paris, France.

88. Institut du Cerveau (ICM), UMR S 1127, Inserm U1127, CNRS UMR 7225, Sorbonne Université, 75013, Paris, France.

89. Univ. Bordeaux, MRGM INSERM U1211, CHU de Bordeaux, Service de Génétique Médicale , F-33000 Bordeaux, France.

90. Laboratoire de Génétique Moléculaire, Service de Génétique Médicale, CHU Bordeaux – Hôpital Pellegrin, Place Amélie Raba Léon, 33076 Bordeaux Cedex, France.

91. Institute of Rare Diseases Research, Spanish Undiagnosed Rare Diseases Cases Program (SpainUDP) & Undiagnosed Diseases Network International (UDNI), Instituto de Salud Carlos III, Madrid, Spain.

92. Molecular Genetics and Functional Genomics, Ospedale Pediatrico Bambino Gesù, IRCCS, Rome, Italy.

93. Med Biotech Hub and Competence Center, Department of Medical Biotechnologies, University of Siena, Italy.

94. Medical Genetics, University of Siena, Italy.

95. Genetica Medica, Azienda Ospedaliero-Universitaria Senese, Italy.

96. Institute of Genomic Medicine and Rare Diseases, Semmelweis University, Budapest, Hungary.

97. Clinical Institute of Genomic Medicine, University Medical Centre Ljubljana, Slovenia.

98. Institute of Systems Motor Science, University of Lübeck, Ratzeburger Allee 160, 23562, Lübeck, Germany.

99. Institute of Neurogenetics, University of Lübeck, Ratzeburger Allee 160, 23562, Lübeck, Germany.

100. Department of Neurology, University Hospital Schleswig Holstein, Ratzeburger Allee 160, 23562, Lübeck, Germany.

101. Pediatric Neurology Research Group, Vall d’Hebron Research Institute, Universitat Autònoma de Barcelona, Barcelona, Spain.

102. Institute of Neuroscience, Universitat Autònoma de Barcelona, Barcelona, Spain.

103. Diagnostic Immunology Research Group, Vall d’Hebron Research Institute (VHIR), Barcelona, Spain.

104. Immunology Division, Genetics Department. Vall d'Hebron University Hospital (HUVH), Barcelona, Spain.

105. Infection in Immunocompromised Pediatric Patients Research Group, Vall d’Hebron Research Institute (VHIR), Barcelona, Spain.

106. Pediatric Infectious Diseases and Immunodeficiencies Unit, Vall d’Hebron University Hospital (HUVH),Barcelona, Spain.

107. Immunology Unit. Department of Cell Biology, Physiology and Immunology. Autonomous University of Barcelona (UAB), Bellaterra, Spain.

108. Neuromuscular Disorders Unit , Department of Pediatric Neurology. Hospital Sant Joan de Déu, Barcelona, Spain

109. Department of Neuropediatrics and Muscle Disorders, Medical Center, Faculty of Medicine, University of Freiburg, Freiburg, Germany.

110. Centro Nacional de Análisis Genómico (CNAG-CRG), Center for Genomic Regulation, Barcelona Institute of Science and Technology (BIST), Barcelona, Spain.

111. Children's Hospital of Eastern Ontario Research Institute, University of Ottawa, Ottawa, Canada.

112. Institute for Immunodeficiency, Center for Chronic Immunodeficiency (CCI), Medical Center, Faculty of Medicine, Albert-Ludwigs-University of Freiburg, Germany.

113. Clinic of Rheumatology and Clinical Immunology, Center for Chronic Immunodeficiency (CCI), Medical Center, Faculty of Medicine, Albert-Ludwigs-University of Freiburg, Germany.

114. DZIF – German Center for Infection Research, Satellite Center Freiburg, Germany.

115. CIBSS – Centre for Integrative Biological Signalling Studies, Albert-Ludwigs University, Freiburg, Germany.

116. RESIST – Cluster of Excellence 2155 to Hanover Medical School, Satellite Center Freiburg, Germany.

117. Nuffield Department of Clinical Neurosciences, University of Oxford, UK.

118. Folkhälsan Research Centre and Medicum, University of Helsinki, Helsinki, Finland.

119. Tampere Neuromuscular Center, Tampere, Finland.

120. Vasa Central Hospital, Vaasa, Finland.

121. Department of Clinical Neurosciences, University of Cambridge, Cambridge, UK.

122. Medical Research Council Mitochondrial Biology Unit, University of Cambridge, Cambridge, UK.

123. Department of Paediatrics, University of Cambridge, Cambridge, UK.

124. East Anglian Medical Genetics Service, Cambridge University Hospitals NHS Foundation Trust, Cambridge, UK.

125. Bellvitge Biomedical Research Institute (IDIBELL), Barcelona, Spain.

126. Medizinische Klinik und Poliklinik IV – Campus Innenstadt, Klinikum der Universität München, Munich, Germany.

127. MGZ - Medical Genetics Center, Munich, Germany.

128. Institute of Clinical Genetics, University Hospital Carl Gustav Carus, Technical University Dresden, Dresden, Germany.

129. Center for Personalized Oncology, University Hospital Carl Gustav Carus, Technical University Dresden, Dresden, Germany.

130. Koç Universıty,School of Medicine, Translational Medicine Research Center, KUTTAM-NDAL Istanbul Turkey.

131. Dpt. of Neurology, Ghent University Hospital, Ghent, Belgium.

132. Program for Undiagnosed Rare Diseases (UD-PrOZA), Ghent University Hospital, Ghent, Belgium.

133. Center for Medical Genetics, Ghent University Hospital, Ghent, Belgium.

134. Department of Biomolecular Medicine, Faculty of Medicine and Health Sciences, Ghent University, Ghent, Belgium.

135. Neuroscience Department, Children's Hospital A. Meyer-University of Florence, 50139, Florence, Italy.

136. Department of Clinical and Experimental Epilepsy, UCL Queen Square Institute of Neurology, and Chalfont Centre for Epilepsy, Gerrard Cross, UK.

137. Department of Neurology, University Hospitals Leuven, Leuven, Belgium.

138. Laboratory for Muscle Diseases and Neuropathies, Department of Neurosciences, and Leuven Brain Institute (LBI), KU Leuven - University of Leuven, Leuven, Belgium.

139. Department of Clinical Genetics, Leiden University Medical Center, Leiden, The Netherlands.

140. Ludwig Boltzmann Institute for Rare and Undiagnosed Diseases, Vienna, Austria.

141. St. Anna Children’s Cancer Research Institute (CCRI), Vienna, Austria.

142. CeMM Research Center for Molecular Medicine of the Austrian Academy of Sciences, Vienna, Austria.

143. Department of Pediatrics and Adolescent Medicine, Medical University of Vienna, Vienna, Austria.

144. St. Anna Children's Hospital, Department of Pediatrics and Adolescent Medicine, Medical University of Vienna, Vienna, Austria.

145. Centre de Génétique Humaine, Institut de Pathologie et de Génétique, Gosselies, Belgium.

146. Département de Médecine, Université de namur (Unamur), Namur, Belgique.

147. Department of Neurology, Klinikum rechts der Isar, Technical University Munich, Munich, Germany.

148. Neurology / Neurogenetics Laboratory University of Crete, Heraklion, Crete, Greece.

149. Aristotle University of Thessaloniki, Thessaloniki, Greece.

150. 1st Department of Neurology, Aristotle University of Thessaloniki, University General Hospital of Thessaloniki, AHEPA, Thessaloniki, Greece.

151. Neurochemistry and Biomarker Unit, 1st Department of Neurology, School of Medicine, National and Kapodistrian University of Athens, Eginition Hospital, Athens, Greece.

152. Pediatric Neurology and Muscular Disease Unit, IRCCS Istituto Giannina Gaslini, Genoa, Italy.

153. Department of Neurosciences, Rehabilitation, Ophthalmology, Genetics, Maternal and Child Health, University of Genoa, Genoa, Italy.

154. Unit of Medical Genetics, IRCCS Istituto Giannina Gaslini, Genoa, Italy.

155. Clinical Bioinformatics, IRCCS Istituto Giannina Gaslini, Genoa, Italy.

156. Pediatric Neurology Department, Saint-Luc University Hospital, Université Catholique de Louvain, Brussels, Belgium.

157. Neurology Department, Erasme Hospital, Université Libre de Bruxelles , Bruxelles, Belgium.

158. Institute of Pathology and Genetics, Charleroi, Belgium.

159. Human Genetics Department, Saint-Luc University Hospital, Université Catholique de Louvain, Brussels, Belgium.

160. Institute of Interdisciplinary Research in Human and Molecular Biology, Human Genetics, IRIBHM, Université Libre de Bruxelles, Brussels, Belgium.

161. Institute of Human Genetics, University Hospital Essen, University Duisburg-Essen, Essen, Germany.

162. Institut du Cerveau et de la Moelle épinière (ICM), Sorbonne Université, UMR S 1127, Inserm U1127, CNRS UMR 7225, F-75013 Paris, France.

163. Department of Pediatric Neurology, Developmental Neurology and Social Pediatrics, Children's Hospital University of Essen, Essen, Germany.

164. Department of Neurology, Heimer Institute for Muscle Research, University Hospital Bergmannsheil, Ruhr-University Bochum, 44789 Bochum, Germany.

165. Luxembourg Centre for Systems Biomedicine, University of Luxembourg, Esch-sur-Alzette, Luxembourg.
